# The Effect of Different Auditory Conditions on Pattern-Reversal Visual Evoked Potentials (PVEPs) in Healthy Adults

**DOI:** 10.3390/brainsci16101115

**Published:** 2026-10-15

**Authors:** Kholoud Ahmad Bokhary, Weaam Abdullah Alrashed, Fahad Ali Algahtani, Mansour Mohammad Almufarrej, Murad Omar Almomani, Hala Khalid Jamous, Abdulrahman Faisal Albloushi, Hanan Khalid Mofty

**Affiliations:** 1 Department of Optometry, College of Applied Medical Sciences, King Saud University, Riyadh 11433, Saudi Arabia; 443202788@student.ksu.edu.sa (W.A.A.); hmofty@ksu.edu.sa (H.K.M.); 2 Ophthalmology Department, College of Medicine, King Saud University, Riyadh 12372, Saudi Arabia; alqfahd@ksu.edu.sa (F.A.A.); aalbloushi1@ksu.edu.sa (A.F.A.); 3 Ophthalmology Department, King Abdulaziz Medical City, Riyadh 11426, Saudi Arabia; almufarrej@hotmail.com; 4 Audiology and Balance Department, College of Medicine, King Saud University, Riyadh 12372, Saudi Arabia; mualmomani@ksu.edu.sa; 5 College of Medicine, King Saud Bin Abdulaziz University for Health Sciences, Riyadh 11426, Saudi Arabia; jamous10344@ksau-hs.edu.sa

**Keywords:** pattern-reversal visual evoked potential (PVEP), auditory conditions, amplitude and latency, human and health

## Abstract

**Highlights:**

**What are the main findings?**

- The baseline (silence) produced higher amplitudes than Quran recitation and music exposure at the 1° stimulation size in healthy adults.
- Stimulation size may influence the sensitivity of PVEP in detecting functional differences in the visual pathway, with significant correlation between BCVA and P100 amplitude observed during Quran recitation at 0.25°.

**What are the implications of the main findings?**

- The findings suggest that the auditory environment must be standardized and reported during PVEP recording.
- The association between visual acuity and P100 amplitude during music exposure suggests that auditory conditions may interact with visual function when using small visual stimuli.

**Abstract:**

**Background/Objectives**: The aim of this study was to investigate the effect of different auditory conditions on pattern-reversal visual evoked potential (PVEP) responses among healthy adults. **Methods**: This repeated-measure design with a fixed baseline and randomized order of the two auditory conditions was conducted at the Retina Clinic, Ophthalmology Department, Tertiary Hospital in Riyadh, Saudi Arabia. Healthy adults with no systemic or ocular diseases aged 20–60 years were included. All participants underwent comprehensive visual and ocular assessment, including best-corrected visual acuity (BCVA), slit-lamp examination, optical coherence tomography (OCT), screening audiometry, and a PVEP test conducted under three auditory conditions: silence, Quran recitation, and music exposure. P100 latency and amplitude were recorded using 1° and 0.25° stimulation sizes. Statistical analysis was performed using non-parametric statistics. A *p*-value of less than 0.05 was considered statistically significant. **Results**: Significant differences in PVEP amplitude responses were observed across auditory conditions, particularly at the 1° stimulation size. The Friedman test demonstrated significant amplitude differences at the 1° stimulation size (χ^2^ = 16.379, *p* < 0.001), with silence producing adjusted significantly higher amplitudes compared with Quran recitation and music exposure (*p* = 0.001 and *p* = 0.003), respectively. Significant positive correlations were identified between BCVA and P100 amplitude at Quran exposure (r = 0.178, *p* < 0.05) only at the 0.25° stimulation size. **Conclusions**: The stimulus size can be sensitive in revealing auditory-related changes in different retinal areas of functional visual pathway alterations. In comparison to baseline (silence), which generally produces the highest amplitudes, both auditory conditions (Quran recitation and music) were associated with lower P100 amplitude, while no significant difference was observed between Quran recitation and music.

## 1. Introduction

Pattern-reversal visual evoked potential (PVEP) is a sensitive non-invasive electrophysiological measure of visual pathway function from the retina through the optic nerve to the occipital cortex in the brain, elicited by visual stimulation recorded from scalp electrodes placed over the occipital cortex [1,2]. These signals are transmitted through bipolar and ganglion cells, where axons converge to form the optic nerve [3].

The gold standard and most widely used type in clinical and research settings for evaluating optic nerve function is PVEP [4]. It produces a specific waveform consisting of three major components: an initial negative deflection at approximately 75 ms (N75), a prominent positive peak at approximately 100 ms (P100), and a subsequent negativity at approximately 145 ms (N145). The P100 component serves as the primary clinical indication of visual pathway stimulation because of its relative stability for many individuals [4]. The component of P100 displays corresponding neural activity across the primary visual cortex (V1) and early extrastriate areas. The latency of P100 is affected by signal transmission speed along the visual pathway, whereas the amplitude depends on the synchrony and degree of the cortical response. Normative P100 latencies in healthy adults usually range from 95 to 115 ms, and amplitudes from 5 to 15 µV [5]. The normal values for P100 latency and amplitude, with a range of physiological and environmental variables such as age, gender, visual acuity, electrode placement, and stimulus parameters, can influence these parameters [5].

Research interest concerns the influence of simultaneous auditory input on PVEP characteristics. The type of sound present and its differing rhythm, complexity, or emotional tone during PVEP recording or visual task performance can influence PVEP amplitude and latency parameters [6,7]. A multisensory combination of visual and auditory input is a fundamental feature of cortical organization of the brain, as it does not process sensory modalities in strict isolation [8]. Auditory signals can reach the visual cortex via corticocortical networks and can transform the neural activity applied to incoming visual information [9]. This modulation depends on the type of auditory stimulus presented, whether it is silence, speech, music, noise, or emotionally balanced sound [10,11]. Thus, understanding the relationships between vision and audio has consequences for multisensory neuroscience and PVEP records, which can serve in the development of auditory-assisted visual rehabilitation strategies. Manjarrez et al. [12] reported an inverted U-shaped relationship between mechanical noise and the P100 VEP amplitude, in which noise from one sensory modality enhances the evoked response [12]. A study showed that classical music had no significant effect on VEP amplitude or latency compared to silence, indicating that classical auditory input may not interfere with visual cortical processing [13]. These findings suggest that background music can be used during VEP recording without compromising signal quality. Another study investigated the effect of auditory stimuli consisting of simple tones and visual stimulation during object recognition in healthy subjects. It found that visual cortical activation was increased within 40–90 ms in early event-related potentials, with increased P100 and reduced amplitudes observed when visual stimuli were paired with congruent sound, indicating early cross-modal integration and facilitated visual processing [14].

Moreover, noise had general amplitude-changing effects; the semantics and characteristics of sound exert distinct influences on early visual cortical processing. Cross-modal congruency, represented by the degree of matching between auditory and visual stimuli in meaning or context, is a key determining factor of how auditory input changes visual responses. Molholm et al. [8] examined cross-modal combination using event-related potentials (ERPs) elicited by auditory (noise bursts), visual (flashes), and simultaneous audiovisual stimuli. They identified early neural interactions within the first 200 ms post-stimulus onset, consistent with rapid audiovisual convergence near primary sensory cortices. It was reported that bimodal stimuli facilitated faster and more accurate responses than unimodal stimuli, and the neural response to combined stimulation exceeded the sum of the individual modality responses earlier, demonstrating good multisensory enhancement [8].

Speech can also be associated with audiovisual integration at the cortical level. Research on auditory–visual speech combinations highlighted that even speech sounds can reduce the amplitude of the visual event-related potential when presented in close temporal proximity to visual stimuli [15]. The N100 auditory-evoked component is significantly shorter in latency when matching audiovisual speech stimuli are presented compared to either unimodal auditory speech or different audiovisual combinations. This suggests that the semantic and phonological content of speech shapes early neural synchronization across auditory and visual cortices [16].

The effect of auditory conditions on PVEP is under investigation through mechanisms including multisensory stochastic resonance, cross-modal audiovisual combination, different categories of sound ranging from music to noise, and speech, which exert differential effects on VEP amplitude and latency. This study aimed to investigate the effect of different auditory conditions (Quran recitation and classical music) on PVEP responses among healthy adults by exploring the interaction of visual neurophysiology and auditory conditions, highlighting the potential role of auditory stimuli in modulating visual cortical responses, and demonstrating cross-modal integration between sensory systems [7]. This study investigated the effect of different auditory conditions on PVEP responses among healthy adults. This study provides novel insight into how auditory input interacts with visual neurophysiology, offering an objective, non-pharmacological and supportive approach—especially in populations with limited communication abilities.

## 2. Materials and Methods

**Figure 1:**
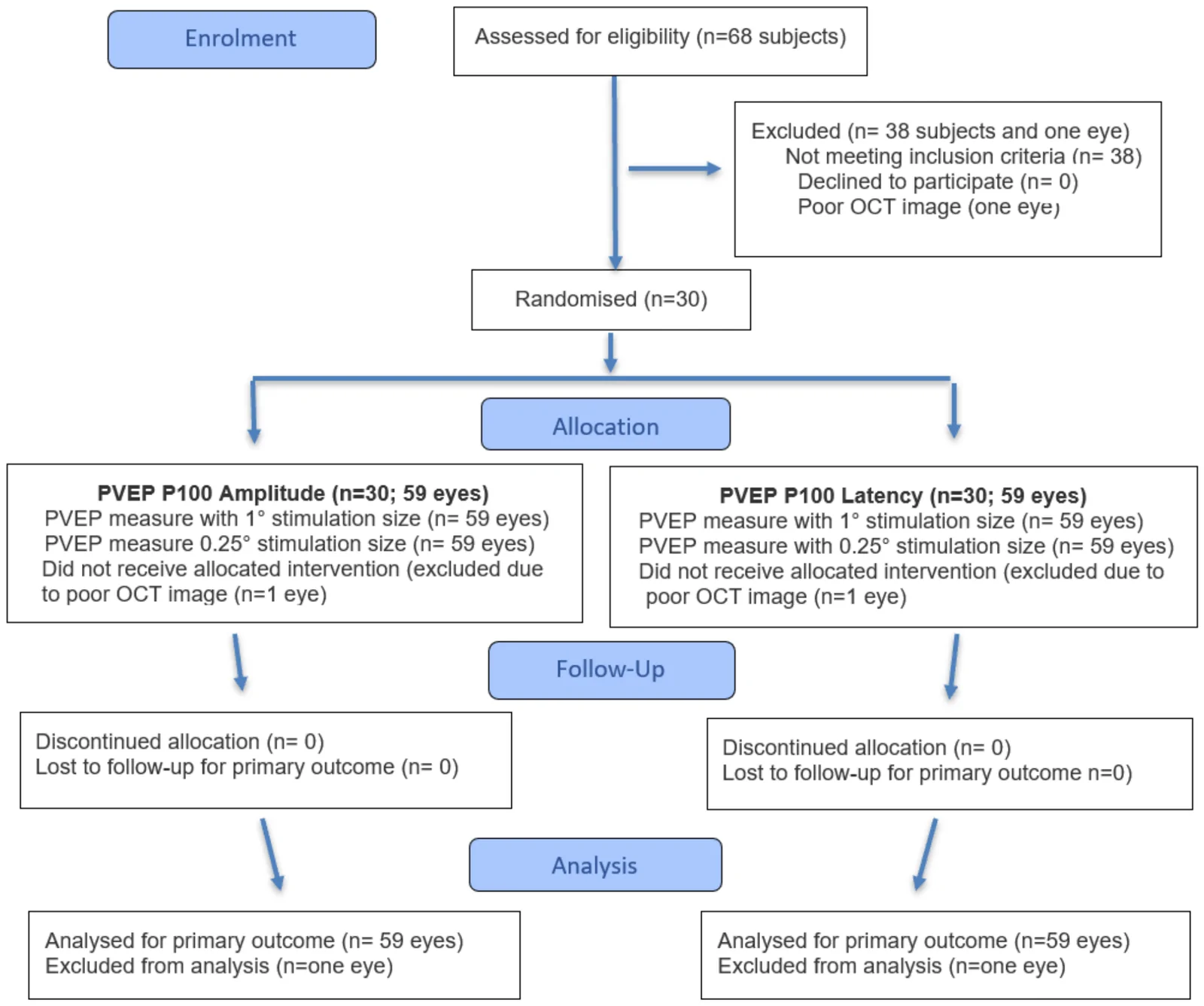
CONSORT flow diagram of the participants through the phases of a randomized trial of enrolment, intervention allocation, follow-up, and data analysis.

This repeated-measure design with a fixed baseline and randomized order of the two auditory conditions was conducted at the retina clinic in the Ophthalmology Department at King Abdulaziz University Hospital, Riyadh, Saudi Arabia. The study was designed and reported in accordance with the CONSORT guidelines. The participant recruitment, allocation, follow-up, and analysis processes are summarized in the flow diagram presented in Figure 1. Ethical approval was obtained from the institutional review board (IRB) and the ethics scientific research committee at King Saud University (Approval No. E-25-10435; approval date: 1 January 2026). The experiments in this study were conducted from the period between January and June 2026. The study was registered at the National Library of Medicine, ClinicalTrials.gov (RCT registration no: NCT07850245, registration date 24 September 2026).

Healthy subjects aged between 20 and 60 years and with no ocular or systemic diseases were included in this study. Subjects with best corrected visual acuity (BCVA) of 0.40 LogMAR or better and with normal hearing were included; subjects aged under 20 or over 60 were excluded to minimize the influence of age-related variability in PVEP latency and amplitude. Subjects with ocular disorders or any neurodegenerative disorders, hearing impairment, or best visual acuity less than 0.40 LogMAR were excluded from this study due to the impact on visual stimulation [17]. Patients taking neurotoxic drugs were also excluded as these conditions can independently affect neural transmission, cortical responses, or the accuracy of visual and auditory evoked potential recording.

A total of 30 participants (60 eyes) were included in this study. Based on previous PVEP studies, it was reported that patients had significantly prolonged P100 latency and amplitude. A sample size of 30 was calculated using the formula for mean difference, two-sided test with an alpha error of 0.05 and a power of 80.0% [18]. To cover both outcomes of prolonged P100 latency and amplitude and allow for potential data loss due to artifacts, this study aimed to recruit about 50 eyes.

Measurements including best-corrected Visual Acuity (BCVA), slit-lamp examination, PVEP test to measure P100 latency and amplitude with two check sizes (1° and 0.25° visual angle), OCT test, and screening audiometry were performed. Considering differences in refractive errors and visual acuity between each eye of the participants, all recordings were performed monocularly, with the right and left eyes tested separately. All eyes contributed data under every condition. Although the PVEP response originated from the same occipital cortex. However, other factors have been considered that may affect visual functions (i.e., refractive error and visual acuity), not the occipital cortex. As refractive errors and BCVA may differ between the two eyes, each eye was treated as a separate observation while accounting for the repeated measures nature of the data. Thus, eye-specific factors justified monocular analysis while the cortex and electrode montage are unchanged.

Visual acuity was assessed using the Early Treatment Diabetic Retinopathy Study (ETDRS) chart and recorded in a LogMAR unit. Slit-lamp examination was performed to evaluate ocular health, particularly retinal status. OCT examination was performed using Spectralis SD-OCT Heidelberg Engineering (Inc., Heidelberg, Germany) to detect ocular conditions and measure central macular thickness (CMT) and ganglion cell layer (GCL) thickness. The P100 peaks of PVEP were measured using the Espion Diagnosys system automated software device (manufacture name: Diagnosys LLC, located in Lowell, MA, USA, version no. V7.1.1). For labeling of P100, the automatically detected peaks were manually checked. The order of the two stimulus check sizes (1° and 0.25° visual angle) was fixed, with the 1° stimulus check size used first and then the 0.25° stimulus check size.

Hearing assessment was performed using pure-tone audiometry at the audiology clinic to evaluate hearing status. The test was conducted using pure-tone audiometry (PTA) (Madsen Astera, manufactured by GN Otometrics/Natus, Taastrup city, Denmark). Pure-tone hearing thresholds were assessed separately for the right and left ears at Hz. Hearing levels were recorded in dB HL. For each participant, the mean threshold across tested frequencies was used as the auditory variable, and that value was assigned to both eyes in the eye-level correlation analysis. All participants had normal hearing, defined as a pure-tone threshold of 20 dB HL or better in each ear.

### Procedure

All participants read the study information and signed the consent form. After obtaining the BCVA and checking ocular health via a macular OCT test, hearing assessment was performed to confirm the inclusion criteria of subjects. For the PVEP test, participants were seated at a fixed distance of 1 m from the stimulus monitor under light-adapted conditions. Each eye was tested separately while the participant wore their BCVA correction in place to ensure optimal visual performance during recording. The fellow eye was occluded during monocular stimulation. Patients with presbyopia require an additional +1.00 Diopter for optimal focus at a 1 m viewing distance and +2.00 Diopter at a 50 cm viewing distance. Scalp electrodes were positioned according to the international 10-10 system. The active electrode was placed on the occipital scalp (Oz), the reference electrode at the mid-frontal position (Fz), and the ground electrode at the vertex (Cz). The impedance of passive skin electrodes measured between 10 and 100 Hz should normally be 5 kΩ or less to ensure high signal quality and reduce electrical noise.

The contrast ratio of the black and white checkerboard PVEP stimulus was 1:1500 (100%), and the luminance of the stimulus monitor was set at 100 cd/m^2^ in accordance with the International Society for Clinical Electrophysiology of Vision (ISCEV) standard [4]. For each eye, two consecutive recordings were obtained using two check sizes (1° and 0.25° visual angle), pattern-reversal checkerboard stimulation with a check size of the standard 60 and 15 arc-minute checks, and continuous acquisition for approximately 15 min, incorporating real-time artifact rejection and signal averaging to enhance waveform stability.

Each auditory condition assessment required approximately 20 min and a 5 min break, resulting in a total experimental duration of about 75 min per participant. This protocol was repeated under three auditory conditions delivered via calibrated speakers at a moderate sound intensity of 65 dB SPL and a volume level of 19. The test was conducted under three conditions: baseline (silence) and two auditory conditions (Quran and music). Quran and music conditions were introduced randomly after silence in order to minimize potential order effects during testing. The primary outcomes included P100 latency (ms) and P100 amplitude (µV) recorded for both check sizes (1° and 0.25°) and averaged per condition and eye [13].

Data were analyzed using the Statistical Package for the Social Sciences (SPSS) version 22. Descriptive statistics were used to summarize demographic and clinical characteristics of the participants and were presented as mean ± standard deviation (SD) for continuous variables and frequencies with percentages for categorical variables. The normality of data distribution was assessed using the Shapiro–Wilk test. Since the PVEP data were not normally distributed, non-parametric statistical methods were applied. The Friedman test was used to compare PVEP, amplitude, and latency across the three auditory conditions (silence, Quran recitation, and music). Bonferroni-corrected post hoc pairwise comparisons were subsequently performed to identify significant differences between auditory conditions. Comparisons between the two check sizes (1° and 0.25°) were also evaluated. The effects of different auditory conditions on PVEP responses were evaluated, and comparisons were performed for both P100 amplitude and latency using 1° and 0.25° check sizes. The order of the two auditory conditions (Quran and music) was randomized and introduced after silence to minimize potential order effects during testing. Spearman’s correlation coefficients for relationships with BCVA, auditory threshold, CMT, GCL, age, and gender were calculated. Inter-eye correlation was accounted for using generalized estimating equations (GEE), with participant eye specified as the clustering variable. As both eyes of participants were included in the analysis, generalized estimating equations (GEE) were used to account for the within-participant correlation between right and left eyes. Separate GEE models were fitted for each PVEP outcome, with the relevant P100 amplitude as the dependent variable. Eye (right versus left) was included as a within-subject factor, and age and pure-tone condition were included as covariates to adjust for their potential effects on P100 amplitude. The association between the explanatory variables and P100 amplitude was evaluated using regression coefficients (B). Adjusted associations of variable parameters with PVEP amplitude under different auditory conditions at 1° and 0.25° stimulations were performed with corresponding 95% confidence intervals (CIs). Statistical significance was defined as a two-sided *p*-value < 0.05.

## 3. Results

A total of 68 subjects were screened and evaluated for eligibility. A total of 38 subjects were excluded because they did not meet the inclusion criteria (i.e., presence of diabetes). Thus, a total of 30 subjects (59 eyes) completed the study procedure across three auditory conditions and were analyzed. One eye was excluded due to a poor OCT image. The age of participants (mean ± standard deviation) was 25.02 ± 3.467 years. All measurements were collected for each participant during a single visit. Table 1 shows the demographic and clinical characteristics of the study participants.

### 3.1. Comparison of P100 Amplitude Across Auditory Conditions

1° Stimulus vs. 0.25° Stimulation Conditions: Both the 1° and 0.25° stimulation sizes demonstrated a statistically significant difference across auditory conditions (χ^2^ = 16.379, *p* < 0.001) and (χ^2^ = 20.068, *p* < 0.001), respectively (Table 2). However, at the 1° and 0.25° stimulation sizes, the latency responses remained relatively stable across silence, Quran recitation, and music exposure, with only mild variations observed between auditory conditions. At the 0.25° stimulation size, only slight variations in P100 latency responses were observed across auditory conditions. The highest latency response was recorded during Quran recitation, followed by music exposure, whereas the lowest latency value was observed during the silence condition. Overall, latency responses remained relatively stable between auditory conditions (Table 2).

**Figure 2:**
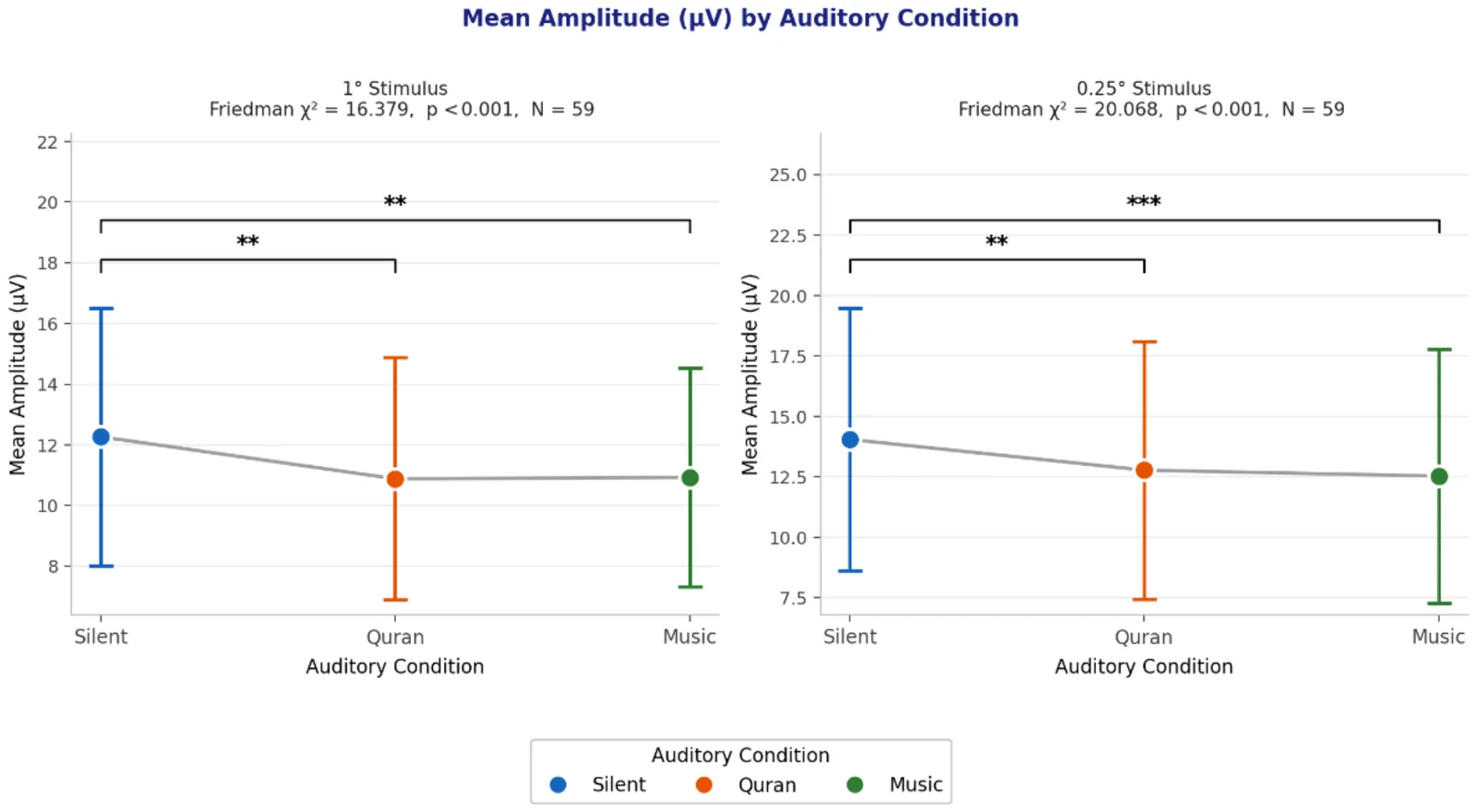
Mean amplitude (µV ± 1 SD) under silent, Quran, and music conditions. (**Left**) panel: 1° stimulus; (**right**) panel: 0.25° stimulus. Brackets indicate statistically significant pairwise differences (Bonferroni-corrected; ** *p* < 0.01, *** *p* < 0.001).

Post hoc pairwise comparisons were carried out with Bonferroni correction at 1° and 0.25° stimulation sizes. At 1°, it was revealed that the silence condition produced significantly higher amplitude responses compared with both the Quran recitation and the music exposure condition. Significant differences were found between the silence condition and both the Quran recitation and music exposure conditions. No statistically significant difference was observed between the Quran recitation and music exposure conditions (Table 3). At 0.25°, it was revealed that the silence condition produced significantly higher amplitude responses compared with both the Quran recitation and the music exposure condition (Table 3). No statistically significant difference was observed between the Quran recitation and music exposure conditions (*p* = 0.1.000) after adjustment. These findings suggest that auditory conditions, regardless of stimulus type, were associated with reduced P100 amplitude responses compared with the silence condition (Figure 2).

### 3.2. Correlation Between P100 Amplitude at 1° and 0.25° Stimulation Sizes and the Clinical Measurements

At 1° stimulation, amplitude showed a weak but significant negative correlation with screening auditory threshold across all auditory conditions, including silence (r = −0.24, *p* < 0.05), Quran (r = −0.259, *p* < 0.05), and music (r = −0.401, *p* < 0.01). No significant correlations were observed with age, gender, BCVA, CMT, or GCL thickness under any auditory condition. At 0.25° stimulation, gender demonstrated a moderate negative correlation with PVEP amplitude in all auditory conditions, including silence (r = −0.471, *p* < 0.01), Quran (r = −0.437, *p* < 0.01), and music (r = −0.353, *p* < 0.01), with female participants being more sensitive in comparison to males. BCVA showed a weak positive correlation with amplitude during the Quran condition only (r = 0.178, *p* < 0.05), whereas no significant association was observed during silence or music. Age, auditory threshold, CMT, and GCL thickness were not significantly correlated with amplitude at 0.25° stimulation under any auditory condition (Table 4).

In the GEE analyses adjusted for eye, age, and auditory thresholds, neither BCVA, CMT, nor GCL thickness was significantly associated with PVEP amplitude at 1° and 0.25° stimulation across the three auditory conditions, as shown in Table 5.

## 4. Discussion

The study investigated the effect of three auditory conditions—silence, Quran recitation, and music—on PVEP responses among healthy adults. Overall, the findings demonstrated that PVEP responses differed across auditory conditions, with the most consistent changes observed in P100 amplitude rather than latency. As this study was designed so that the silence condition was always recorded first as a baseline, while the Quran recitation and music conditions were randomly assigned to the second and third positions, the findings showed that PVEP in the silence condition was higher than that of both auditory conditions, with no difference in PVEPs between the Quran recitation and music conditions, which could be the result of a steady decrease in amplitude throughout the session, even if there were no auditory effects. Thus, the time-dependent decrease was equally shared between the Quran recitation and music conditions, and both remained below their initial values.

More pronounced auditory-related electrophysiological changes were observed at the 1° stimulation size compared with the 0.25° stimulation size, indicating that a larger size of stimulation could be more sensitive for detecting visual pathway changes in normal subjects. A mild correlation was found between P100 amplitude at 0.25° stimulation size and the best-corrected visual acuity (BCVA); however, no significant correlations were identified between retinal thickness and P100 amplitude responses across different auditory conditions.

Previous studies have reported that background classical music does not significantly improve VEP recording quality or reduce recording variability [13]. Another study suggested that auditory conditions and background music may influence cortical processing and attention-related neural activity [19]. In contrast, the present study demonstrated that music exposure was associated with reduced P100 amplitude responses at the 1° stimulation size. The differences between the findings may be attributed to variations in study design and outcome measures, as the previous studies mainly evaluated recording quality and cognitive processing, whereas the present study investigated the influence of auditory conditions on visual cortical electrophysiological responses.

Previous studies have reported abnormal VEP responses in patients with abnormal conditions. For instance, in diabetic retinopathy, there was a particularly prolonged P100 latency and reduced amplitude responses compared with healthy controls [18]. Similarly, another study reported delayed P100 latency and amplitude in patients with diabetes without retinopathy, suggesting that neural dysfunction may occur before clinically detectable retinal changes [17]. This suggests that a study should be conducted using the same current protocol on diabetic subjects to confirm these findings.

The check size appeared to influence the sensitivity of PVEP responses in the present study. More pronounced electrophysiological changes were observed at the 1° stimulation size compared with the 0.25° stimulation size. This finding is consistent with the International Society for Clinical Electrophysiology of Vision (ISCEV) standards for clinical VEP recording, which recommend the use of both large and small check sizes during pattern-reversal stimulation [4]. Previous studies have also demonstrated that check size can significantly influence P100 latency and amplitude responses [20]. Smaller check sizes may provide a more specific assessment of central visual function, but they may therefore be more sensitive in detecting subtle central retinal dysfunction. However, the stronger electrophysiological changes observed at the 1° stimulation size in the present study may reflect a wider area of retinal change in normal adults.

Previous studies have suggested that greater ganglion cell layer thickness is associated with stronger visual cortical amplitude responses. They have reported that retinal ganglion cell structure and visual pathway function can be affected before advanced retinal changes become clinically evident [17]. In addition, studies investigating the relationship between OCT structural measures and electrophysiological responses have shown that reduced ganglion cell or inner retinal thickness is associated with abnormal PVEP responses, including reduced amplitude and delayed latency [21]. Therefore, the positive correlation observed in the present study suggests a relationship between stimulus size and functional visual pathway responses in some macular changes, such as in age-related macular degeneration (AMD) and diabetic retinopathy (DR), which needs more investigation. Gender correlation to P100 amplitude at 0.25° during all auditory conditions could be related to hormones and their influence on auditory sensitivity, especially in premenopausal women [22].

Several neurophysiological mechanisms have been proposed to explain the influence of auditory conditions on PVEP parameters in normal subjects. Corticocortical and thalamocortical pathways connecting auditory and visual processing regions allow rapid bidirectional communication between sensory areas [23]. Anatomical evidence in humans and non-human primates has demonstrated projections from auditory association cortex to visual areas V1 and V2, providing a substrate for direct auditory modulation of early visual responses. In addition, the reticular activating system and thalamocortical arousal networks modulate cortical excitability globally. Engaging or emotionally salient sounds increase arousal and thereby raise the gain applied to incoming sensory input across modalities [24]. This is contradicted by findings that high-arousal sounds produce larger early visual ERP components than low-arousal neutral sounds. Stochastic resonance as a mechanism requires a nonlinear neural system in which an optimal level of background noise lowers the effective threshold for signal detection or response. Neurons in the visual cortex, operating at or near threshold, may be pushed into a response range by concurrent auditory noise, producing enhanced VEP amplitudes at moderate auditory noise levels [25]. The dependence of this effect on noise intensity and its reduction at high noise levels are characteristic of the SR phenomenon and have been demonstrated empirically in auditory–visual paradigms. In our study, the mild correlation between screening auditory threshold and PVEP responses at 1° stimulation size might emphasize the general correlation between PVEP and auditory sensory regions.

The present findings may have important clinical implications for the early detection and monitoring of visual pathway dysfunction in many ocular conditions such as retinopathies, glaucoma, and other retinal dystrophies in adults. Therefore, PVEP testing may contribute to the early identification of visual pathway dysfunction and aid in monitoring disease progression before severe visual impairment develops. The test could be applied as an objective tool that does not rely on subjective reporting, with potential applications in populations with limited communication, such as children, individuals with autism spectrum disorder, and patients with Down syndrome. The findings of this study also highlight the importance of standardization in clinical PVEP practice and open new possibilities for multisensory approaches to visual rehabilitation. Although the effect sizes at 1° and 0.25° were small (Table 2), these differences may still reflect subtle changes in visual cortical processing, so clinical significance should be interpreted cautiously.

Several limitations should be considered in the present study. Individual attentional and emotional responses to auditory stimulation could not be controlled during PVEP recording. The silence condition was always performed first as a baseline measure of PVEP, while the Quran recitation and music conditions were randomized following the silence condition. Given the procedure duration of recordings for each auditory condition and standardized baseline procedure, fatigue effects are expected. It is known that P100 amplitude decreases with prolonged, repeated pattern-reversal stimulation [26]. However, the possibility of a residual sequence influence due to the fixed position of the silence baseline cannot be entirely excluded, and this was considered in this study when interpreting the findings. Another limitation of this study was the use of individual eyes as the unit of analysis, as both eyes from the same participant may not entirely, despite applying appropriate statistical methods to address correlation. It has also acknowledged that the age of participants was narrowed to a young age in comparison to the stated inclusion range of 20–60 years. Thus, the present findings may not be representative of older healthy subjects. The auditory stimuli were presented at a fixed intensity (65 dB SPL) rather than being individually calibrated to each participant’s hearing threshold. However, all participants had normal hearing confirmed by hearing assessment prior to the experiment. Therefore, the standardized sound level was intended to ensure consistency across participants. Nevertheless, individual calibration relative to hearing thresholds could provide a more personalized measure of auditory stimulation and should be considered in future studies. Moreover, although Quran recitation may carry cultural or religious meaning for some participants, the present study did not examine religious or cultural responses to the stimulus. Recitation was used as one type of auditory condition to assess PVEP responses. As the participants’ religious and linguistic backgrounds were not known, generalizability to other populations is unknown. Further studies involving participants from different cultural and religious backgrounds are warranted to confirm generalizability. Despite these limitations, the study provides valuable insights into the effects of auditory conditions on visual cortical responses in normal subjects. Future studies may explore the potential application of auditory-modulated VEP assessment in special clinical populations, such as patients with diabetes, glaucoma, autism spectrum disorder, or non-verbal individuals, where objective electrophysiological evaluation may provide valuable clinical information.

## 5. Conclusions

The stimulus size can be sensitive in revealing auditory-related changes at different retinal areas of functional visual pathway alterations. In comparison to silence, which generally produces the highest amplitudes, both auditory conditions (Quran recitation and music) were associated with lower P100 amplitude, while no significant difference was observed between Quran recitation and music.

## Figures and Tables

**Table 1:** Summary of the demographic and clinical characteristics of the study participants.

	Mean ± Std. Deviation	
N	30 Subjects (59 eyes)	
Age	25.02 ± 3.4	
Gender–Female (%)	34 ± 0.4 (57.6)	
BCVA (LogMAR)	0.019 ± 0.00	
CMT (µm)	267.39 ± 7.5	
GCL (µm)	52.89 ± 2.6	
Pure Tone Audiometry (dB)	4.56 ± 2.5	
P100 Amplitude (µV)	at 1° Stimulation size	at 0.25° Stimulation size
Silence	12.35 ± 4.2	14.05 ± 5.4
Music	10.93 ± 3.6	12.53 ± 5.2
Quran	10.88 ± 3.9	12.78 ± 5.3
P100 latency (ms)		
Silence	91.85 ± 4.8	98.15 ± 4.2
Music	92.20 ± 4.9	98.30 ± 5.0
Quran	91.60 ± 4.0	98.38 ± 5.1

Abbreviations: BCVA = visual acuity; LogMAR = logarithm of the minimum angle of resolution; dB = decibel; CMT = central macular thickness; GCL = ganglion cell layer.

**Table 2:** Analysis of P100 amplitude and latency across auditory conditions at 1° and 0.25° stimulation sizes.

P100 Amplitude (*n* = 59 Eyes)															
1° Stimulation Size								0.25° Stimulation Size							
	Mean	Std. D	Min	Max	Chi-Square	Effect Size	Sig	Mean	Std. D	Min	Max	Chi- Square	Effect Size	Sig	1° vs. 0.25°
Silence (A_S)	12.26	4.23	3.2	21.6	16.379	0.14	<0.001	14.05	5.42	1.6	27.8	20.06	0.17	<0.001	−1.539 *p* = 0.124
Quran (A_Q)	10.88	3.99	1.7	18.7				12.78	5.32	2.6	24.8				−1.558 *p* = 0.119
Music (A_M)	10.93	3.60	2.4	17.6				12.53	5.24	1.8	24.8				−1.589 *p* = 0.112
				P100 Latency (*n* = 59 eyes)											
		1° stimulation size									0.25° stimulation size				
Silence (L_S)	91.85	4.80	82	101	0.442	0.003	0.802	98.15	4.24	95	115	0.542	0.004	0.770	−6.767 *p* < 0.001
Quran (L_Q)	91.60	4.04	85	95				98.38	5.11	95	115				−7.457 *p* < 0.001
Music (L_M)	92.20	4.93	85	110				98.30	5.04	95	115				−6.881 *p* < 0.001

Abbreviations: S = Silence, Q = Quran recitation, M = music exposure, Min = minimum; Max = maximum, Std. Dev = standard deviation. Values are presented as mean latency (ms). Values are presented as mean P100 amplitude (µV).

**Table 3:** Bonferroni-corrected post hoc pairwise comparisons of P100 amplitude across auditory conditions at the 1° and 0.25° stimulation sizes.

	1° Stimulation Size				0.25° Stimulation Size			
Auditory Conditions Comparisons	Mean Difference-Rank	Std. Error	Std. Test Statistic	Adj Sig.	Mean Difference-Rank	Std. Error	Std. Test Statistic	Adj. Sig.
**Quran–Music**	−0.086	0.186	−0.464	1.000	0.068	0.184	0.368	1.000
**Quran–Silence**	0.690	0.186	3.714	0.001	0.746	0.184	4.051	0.000
**Music–Silence**	0.603	0.186	3.250	0.003	0.678	0.184	3.682	0.001

Abbreviations: Adj. Sig = adjusted significance.

**Table 4:** Correlations between different stimulus sizes of PVEP amplitude and participant characteristics under three auditory conditions.

	Amplitude at 1° Stimulation Correlation Coefficient (r)			Amplitude at 0.25° Stimulation Correlation Coefficient (r)		
Auditory Conditions	Silence	Quran	Music	Silence	Quran	Music
**Age**	0.074	−0.7	0.005	−0.113	−0.104	−0.075
***p*** ** value**	0.576	0.598	0.970	0.393	0.434	0.581
**Gender**	0.032	−0.038	0.104	0.471 **	−0.437 **	−0.353 **
***p*** ** value**	0.813	0.776	0.436	<0.001	<0.001	0.006
**BCVA (LogMAR)**	0.04	0.027	0.086	0.104	0.178 *	0.151
***p*** ** value**	0.764	0.838	0.523	0.432	0.177	0.253
**Auditory (dB)**	−0.24	−0.259 *	−0.401 **	−0.148	−0.121	−0.156
***p*** ** value**	0.063	0.048	0.002	0.265	0.362	0.239
**CMT (µm)**	0.078	0.078	0.08	−0.004	−0.076	−0.039
***p*** ** value**	0.558	0.558	0.549	0.975	0.565	0.770
**GCL (µm)**	0.163	0.173	0.196	−0.105	−0.189	−0.074
***p*** ** value**	0.217	0.191	0.140	0.428	0.152	0.578
**OD vs. OS 1°**	−0.041	−0.006	−0.013	−0.095	−0.219	−0.112
***p*** ** value**	0.831	0.974	0.947	0.624	0.253	0.564

Abbreviations: (r) = Spearman’s correlation coefficient; BCVA = visual acuity; LogMAR = logarithm of the minimum angle of resolution; dB = decibel; CMT = central macular thickness; GCL = ganglion cell layer. *p* ≤ 0.05 (*), *p* ≤ 0.01 (**).

**Table 5:** Adjusted associations of visual and retinal parameters with PVEP amplitude under different auditory conditions at 1° and 0.25° stimulations.

Auditory Condition	Predictor	B	95% CI	*p*
**Silent (A_S) at 1° stimulation**	BCVA	5.911	−12.179 to 24.00	0.522
	CMT	0.003	−0.127 to0.132	0.968
	GCL	0.327	−0.066 to 0.720	0.103
**Silent (A_S) at 0.25° stimulation**	BCVA	12.942	−10.760 to 36.644	0.285
	CMT	0.001	−0.202 to 0.204	0.995
	GCL	−0.29	−0.563 to 0.504	0.914
**Quran (A_Q) at 1° stimulation**	BCVA	−0.566	−17.888 to 16.756	0.949
	CMT	−0.018	−0.132 to 0.096	0.757
	GCL	0.275	−0.092 to 0.642	0.142
**Quran (A_Q) at 0.25° stimulation**	BCVA	21.242	−4.946 to 47.429	0.112
	CMT	−0.095	−0.692 to 0.503	0.756
	GCL	−0.155	−0.636 to 0.327	0.529
**Music (A_M) at 1° stimulation**	BCVA	3.886	−10.146 to 17.918	0.587
	CMT	−0.018	−0.137 to 0.101	0.763
	GCL	0.318	−0.028 to 0.665	0.072
**Music (A_M) at 0.25° stimulation**	BCVA	13.571	−13.460 to 40.603	0.325
	CMT	−0.016	−0.197 to 0.164	0.860
	GCL	0.046	−0.415 to 0.508	0.844

Abbreviations: PVEP = pattern-reversal visual evoked potential; BCVA = best corrected visual acuity; CMT = central macular thickness; GCL = ganglion cell layer; CI = confidence interval; B = regression coefficient.

## Data Availability

The datasets generated during and/or analyzed during the current study are available from the corresponding author on reasonable request due to privacy and confidentiality considerations.
